# Cancer survivors’ acceptability and perspectives on engagement with digital cognitive-behavioral therapy for insomnia: a mixed methods perspective

**DOI:** 10.1007/s00520-025-09552-0

**Published:** 2025-05-24

**Authors:** Maria I. Clara, Josephine Hegarty, Annemieke Van Straten, Federica Giudetti, Imke Buekenhout, Irem Koc, Maria C. Canavarro, Ana Allen Gomes

**Affiliations:** 1 Center for Research in Neuropsychology and Cognitive Behavioral Intervention (CINEICC), Faculty of Psychology and Educational Sciences, University of Coimbra, Rua Do Colégio Novo, 3000-115 Coimbra, Portugal; 2 Laboratory of Chronopsychology and Cognitive Systems (ChronCog), University of Coimbra, Coimbra, Portugal; 3 School of Nursing and Midwifery, University College Cork, Cork, Ireland; 4 Vrije Universiteit Amsterdam & Amsterdam Public Health Institute, Amsterdam, The Netherlands; 5 Department of Psychology “Renzo Canestrari”, University of Bologna, Bologna, Italy

**Keywords:** Cancer survivors, Cognitive-behavioral therapy, CBT-I, Insomnia, Oncology

## Abstract

**Purpose:**

Insomnia is one of the most prevalent, persistent, and impairing conditions for which cancer survivors want treatment. However, the evidence-based first-line treatment, cognitive-behavioral therapy for insomnia (CBT-I), is seldom available to cancer survivors. Digital CBT-I improves dissemination, but its impact depends on patients’ acceptance and preferences. We aimed to determine the acceptability of and explore perspectives of digital CBT-I for cancer survivors.

**Methods:**

Responses collected at study exit by 123 cancer survivors (mean age 47.1 years, 95.9% women, 76.4% breast cancer) who completed a digital CBT-I, OncoSleep, were analyzed using mixed methods. The exit survey included quantitative measures of acceptability, and qualitative insights were gathered through a free-response item. Responses were systematically coded and analyzed using deductive and inductive approaches to identify recurrent themes.

**Results:**

The digital CBT-I, OncoSleep, received high ratings for perceived efficacy, satisfaction, helpfulness, usability, likelihood of future use of therapeutic techniques, likelihood of recommendation, and user experience. Patients who showed clinically significant improvements in insomnia severity reported better user experience. Analyses revealed eight themes were relevant for engagement with treatment: clinician support and monitoring, interactive features and ease of use, perceived efficacy of treatment techniques, convenience and non-hospital setting, validation of unmet needs, sleep medication discontinuation, tailored treatment content, and empowerment.

**Conclusions:**

Results suggest digital CBT-I is well-accepted among cancer survivors. Digital CBT-I offers opportunities for treatment engagement.

## Background

A growing population of cancer survivors requires ongoing support to manage persistent physical and psychological side effects related to their cancer and treatment. Insomnia is among the most prevalent, long-lasting, and debilitating symptoms or conditions associated with cancer that affects up to one in two people diagnosed with cancer [1–3]. Typically, insomnia presents as symptoms involving difficulties in initiating and/or maintaining sleep. While other cancer-related symptoms often resolve over time, insomnia rapidly becomes self-reinforcing, and if left inadequately treated, can become a chronic disorder in up to 30% of cancer survivors [1, 4, 5]. Insomnia disorder is characterized by the chronic, impairing, and recurrent experience of insomnia symptoms to the extent that they occur more than 3 days a week and result in impaired daytime functioning or significant psychological distress for at least 3 months [6]. The daytime consequences of insomnia include dysphoric states, impaired cognition, and an exacerbation of cancer-related symptoms such as fatigue and pain [7–9]. Insomnia independently decreases quality of life, is an independent risk factor for the development of physical and mental ill health, and there are indications that it might adversely impact immunity and influence tumor growth and progression [1, 10–12]. This underscores the need for readily accessible, effective treatment.

Insomnia can be effectively treated with cognitive behavioral therapy for insomnia (CBT-I), and CBT-I is universally recommended as first-line treatment for insomnia in general as well as in cancer survivors [7, 13–15]. Despite guideline consensus, access to CBT-I is especially limited for cancer survivors, primarily due to a shortage of trained therapists. Consequently, most survivors with insomnia receive sleep medication, unnecessarily exposing them to the associated risk of harm and side effects [9, 16]. Digitally delivered CBT-I extends the reach of this treatment and has shown to be effective in cancer survivors [9, 14, 17, 18]. Considering its effects and advantages for disseminating guideline-concordant treatment to the growing number of cancer survivors, digital CBT-I is recognized as promising for cancer-related insomnia [14].

The impact of digital CBT-I programs is contingent upon how well treatment is accepted and used by those it aims to help. The adoption, engagement, and effectiveness of health interventions depend on their acceptability, including perceived appropriateness of content and quality of care, and in the context of digital interventions, user experience. Beyond functionality and efficiency, digital health interventions must be intuitive, easy to learn, offer users control over interactions, and be stimulating to maintain engagement [19–21]. Treatment engagement, characterized by patients’ attentiveness, time spent, emotional connection, commitment to the digital intervention, and acceptance of its principles and strategies, is essential for the success of digital CBT-I programs [22, 23]. The more positively the treatment is received by patients, the more likely they are to adhere to and engage with it, and the more effective it becomes. Previous research suggests adapting digital health interventions based on user feedback can increase treatment engagement and the effectiveness of digital CBT-I [24–26]. Understanding the strengths and areas for improvement from a patient-centered perspective is crucial for optimizing treatment, ensuring successful implementation, and maximizing the real-world impact of digital CBT-I.

Although patient preferences influence treatment outcomes, these are very rarely captured or reported in clinical research [7, 27]. The acceptability of digital CBT-I and the experiences of cancer survivors who had undergone this treatment remain largely unexamined. Exploring patients’ attitudes and beliefs toward digital CBT-I is essential for identifying factors that can promote engagement, commitment, and ultimately effectiveness. To address this knowledge gap, this study adopted a patient-level approach to explore the experiences of patients who completed digital CBT-I and their acceptability of treatment, using both qualitative and quantitative methods. Our aim was to examine cancer survivors’ perspectives engaging with digital CBT-I and to assess its acceptability, including perceived efficacy, helpfulness of treatment features, usability, likelihood of future use of treatment techniques, likelihood to recommend treatment, and user experience, as well as its association with treatment effectiveness.

## Methods

### Participants and procedures

Participants in this study were cancer survivors who had received digital CBT-I as part of a randomized clinical trial (RCT) [18]. Eligible participants were aged 18 and over, had completed primary cancer treatment (or were undergoing adjuvant treatment), had at least subclinical insomnia symptoms (defined as an Insomnia Severity Index [ISI; 28] score of ≥ 8), and were proficient in Portuguese. Cancer survivors were recruited through self-referral. The study was promoted online, in print, through media outlets, and via clinics and patient associations [28]. Spanning from 2021 to 2024, the study received Institutional Review Board approval, and informed consent was obtained from all participants prior to enrollment. One hundred fifty-four participants were randomly assigned in a 1:1 ratio to either the digital CBT-I intervention group or a waitlist control group. After a period of 8 weeks from randomization, participants in the delayed treatment condition were provided with access to the digital CBT-I.

Those who completed the intervention were invited to share their experiences with the digital CBT-I, including assessing OncoSleep’s acceptability and offering suggestions for improving its implementation, through a study-exit survey that included both quantitative measures and an open-text question. This evaluation focuses on 123 treatment completers, including participants from both the intervention and the waitlist control group. The completion rate was 79.8%, with time constraints (*n* = 5) and changes in clinical status (*n* = 2) cited as reasons for withdrawal, as well as non-responses to follow-up.

### Intervention

The digital CBT-I program was OncoSleep, a web-based program accessible through an internet browser on a computer or smartphone. The program includes six self-guided weekly modules, as well as weekly clinician support via email. Each module, taking around 35 min to complete, has a specific agenda: psychoeducation and goal setting, relaxation training and sleep hygiene, stimulus control and sleep restriction therapies, cognitive therapy, and relapse prevention. In between sessions, patients had to do “homework” (e.g., sleep restriction). The intervention also includes weekly feedback provided by a licensed psychologist with advanced expertise in CBT-I via email after completing a session. This feedback was aimed at motivating patients to carry out the homework assignments and addressing any potential adherence issues noted by the clinician during the weekly review of the interactive sleep diaries that patients were required to complete throughout the intervention. Patients were encouraged to email the clinician if they encountered difficulties in implementing the strategies, rather than abandoning the treatment, and if they had any questions. The clinician spent 2 h per patient during each intervention course (20 min per patient per session). Treatment was presented as a collaborative process, and the importance of engagement was emphasized throughout the intervention.

Sleep diary data were also used to guide the implementation of behavioral interventions via fully automated algorithms and to generate interactive visual graphics and charts that allowed patients to review their progress weekly. Additional features of OncoSleep included supplementary materials for each weekly module, comprising audio files (e.g., relaxation training exercises), educational handouts, case vignettes with examples from cancer survivors, and printable sleep diaries, mind maps (i.e., thought record), and planners (e.g., results of behavioral experiments).

OncoSleep’s treatment content, based on a well-established protocol [29], is adapted for cancer survivors (Table 1). The intervention content is primarily structured as text, but it also includes interactive quizzes, animations, and audio.

**Table 1:** Adaptations of OncoSleep’s CBT-I components tailored for cancer survivors

CBT-I components	Adaptations for cancer survivors
Psychoeducation	Sleep and circadian education. Education on how cancer, treatments and adjuvant therapies, comorbid conditions and side effects can affect sleep. Strategies to manage common comorbid symptoms (i.e., fatigue, hot flashes, and night sweats) Education on the multifactorial nature of insomnia and CBT-I as an active agent at the brain-behavior interface that improves biological, physiological, cognitive, and emotional factors Education on distinguishing sleepiness from fatigue as a cue to go to bed Wind-down and wake-up routine
Relaxation training	Adapted instructions to accommodate conditions/lesions (e.g., lymphedema). Presented relaxation training also as a strategy to alleviate cancer-related symptoms Included a range of relaxation strategies tailored to the clinical situation (e.g., use alternative techniques if progressive muscle relaxation is unsuitable for some patients)
Stimulus control and sleep restriction therapies	Restricted time in bed to the average number of hours slept in the previous week, gradually extended when sleep efficiency cutoff of 85% was reached. Attended to clinical complexities Encourages patients to go out of bed to another room if unable to sleep. Encourages making other places comfortable to rest so that bed is reserved for sleep and sexual activity only Encouraged patients to contact their clinician if they encounter difficulties with the placement of sleep window/adherence to these therapies Reassured patients that these techniques are safe and effective for cancer survivors
Cognitive therapies	Cognitive restructuring (i.e., thought record, experiments) and defusion. Addressed cancer worry and beliefs about the impact of insomnia on health and risk of cancer recurrence, as well as about cancer-related symptoms (e.g., cancer-related fatigue, pain, night sweats) Worry time; discretionary cognitive strategies (i.e., paradoxical intention, cognitive control) Addressed behavior change, barriers to adherence, particularly to behavioral therapies
Additional strategies	Behavioral activation and value-based planning/committed action. Acceptance-based strategies (i.e., willingness to accept the time-limited discomfort of nighttime awakenings/CBT-I strategies and other intrusive and suffering-causing cancer-related cognitions)

### Outcome variables and measures

Patients who completed the OncoSleep intervention were invited to complete an exit survey on their experience with digital CBT-I, which included a qualitative item soliciting free-response feedback: “We would like to hear about your experience with OncoSleep. What did you like best and least about the program? Do you have suggestions for improving the use of this digital intervention?”. The exit questionnaire also included quantitative questions on key dimensions of acceptability: perceived efficacy, helpfulness of treatment characteristics, usability, likelihood of future use of treatment techniques, likelihood to recommend treatment, satisfaction, and user experience (Table 2). Consistent with previous studies, satisfaction with the digital CBT-I was assessed using an adaptation of the Treatment Satisfaction Scale, based on the Consumer Report Treatment Satisfaction Scale [30–32]. Participants were asked, “How much do you feel the OncoSleep has helped you in the following areas?”. A total perceived efficacy score was computed by calculating the mean of all items. The User Experience Questionnaire – Short version (UEQ-S; [33]) was used to assess user experience across two subscales: pragmatic and hedonic quality (4 items each). Pragmatic quality reflects functional aspects related to task efficiency and goal achievement (scale consistency was Cronbach’s alpha (*α*) = 0.88). The hedonic quality captures experiential aspects, including novelty and stimulation (*α* = 0.90). An overall UEQ-S score reflecting the general user experience was also calculated.

**Table 2:** Summary of acceptability measures, scoring criteria, and interpretation

Variable/item name (source of items)	Number of items; details of questions asked	Scoring/analysis	Interpretation of scores
Perceived efficacy (Treatment satisfaction scale; Manber et al., 2011 [31])	Perceived efficacy of intervention in making things better/or not in six areas/items: well-being, coping with stress, energy levels, mood, insomnia, and performance	Responses were rated on a 5-point Likert scale, from “Made things a lot worse” = 0, to “Made things lot better” = 4	Higher scores mean the intervention made things better in the area identified (higher perceived efficacy)
Helpfulness of treatment features	Three scale items assessing the perceived helpfulness of the digital intervention features: digital format, clinician support, and supplementary materials	4-point Likert scale, from “Not helpful at all” = 0 to “Very helpful” = 3	Higher scores indicate the identified feature was considered more helpful
Usability	One item assessing the ease of navigating the program	4-point scale (“Very low” = 0 to “Very high” = 3)	Higher scores suggest the digital intervention was considered more user-friendly
Likelihood of future use of treatment techniques	One item assessing the probability of using the treatment techniques in the future		Higher scores reflect a stronger intention among participants to use the treatment techniques in the future
Likelihood to recommend	One item assessing the probability of recommending the treatment to a friend with the same problem		Higher scores suggest that participants are more likely to recommend the treatment to a friend with insomnia
Overall satisfaction	One item assessing the extent of satisfaction with the digital intervention	4-point scale (“Very dissatisfied” = 0 to “Very satisfied” = 3)	Higher scores reflect greater satisfaction and enjoyment of the digital intervention
User experience (User Experience Questionnaire – Short version [33])	Eight scale items assess two dimensions: pragmatic quality (functionality-focused) and hedonic quality (engagement-focused). Pragmatic quality items include understandability, ease of use, efficiency, and control. Hedonic quality items include excitement, enjoyment, inventiveness, and cutting-edge	7-point Likert scale, ranging from − 3 (fully agree with negative term) to + 3 (fully agree with positive term)	Higher scores in pragmatic quality indicate that users find the digital intervention easier to use and better designed for completing tasks. In hedonic quality, higher scores reflect a more positive emotional experience, with the digital intervention being perceived as more enjoyable and engaging. An overall score of 2.5 or above generally indicates a positive user experience, where the digital intervention is perceived as both usable and enjoyable

For the RCT, all participants completed pre- and post-intervention questionnaires to assess the effectiveness of the intervention, including the Insomnia Severity Index (ISI) [34]. The ISI consists of 7 items, with total scores ranging from 0 to 28, where higher scores indicate more severe insomnia symptoms.

### Statistical methods

Descriptive statistics were computed using IBM SPSS v.29 to characterize participant demographics and the acceptability of the treatment features in the total sample. Patient-level responses for satisfaction/perceived efficacy, helpfulness, usability, likelihood of future use, likelihood to recommend, and enjoyability are reported as percentages. For the UEQ-S, means, standard deviations (SD), and confidence intervals (CI) were calculated for the subscales as well as the overall scale. In the UEQ-S, a SD greater than 1.01 signifies low agreement among users, SD between 0.83 and 1.01 indicates medium agreement, and SD below 0.83 signifies high agreement. Chi-square tests were conducted in the subsample of participants initially allocated to the treatment condition in the RCT to compare user experience scores between participants who experienced clinically significant improvements in insomnia severity (defined as change ≥ 8 in ISI scores from pre- to post-treatment) and those who did not.

For the free text answers, we used thematic analysis following the approach outlined by Braun and Clarke [35]. A hybrid inductive-deductive thematic approach was performed, with a multistage inductive coding process to remain open to tangential and emergent codes. We remained mindful of our positionality and embedded reflexivity throughout the research process. Data was analyzed by researchers from different cultural backgrounds and areas of expertise, with some having personal familiarity or lived experience with the challenges faced by these patients, which informed our reflections and interpretations. By combining both insider and outsider perspectives, we analyzed responses without the influence of our lived experiences while leveraging our shared experiences. Initially, two coders immersed themselves in the data to gain familiarity and systematically coded it by identifying and labeling key features, while maintaining reflexivity notes throughout the coding process. A thematic framework was developed based on research questions and relevant literature [23]. Coders reviewed data independently and then collaboratively discussed their codes and created and refined themes to ensure consistency and alignment. This involved combining predefined and emergent themes through a negotiated process. Finally, two additional coders reviewed the themes to confirm their coherence. NVivo software [36] was used to organize, systematically analyze, and code the patients’ responses and extract data relevant to each theme.

## Results

### Participants

Participants (*N* = 123) had an average age of 47.1 years (SD = 9.9, range: 24–75). The majority were women and most had attained higher education (69.9%; Table 3). Most participants rated their digital skills as good or very good (86.3%).

**Table 3:** Sociodemographic characteristics of participants (*N* = 123)

Characteristic – % (*n*)	
Age group	
24–40	24.4 (30)
41–50	39.0 (48)
51–60	26.8 (33)
61–75	9.8 (12)
Women	95.9 (118)
Highest level of education	
Basic education	3.3 (4)
Secondary education	26.8 (33)
Bachelor	48.8 (60)
Master	17.0 (21)
PhD	4.1 (5)
Occupational status	
Working	53.7 (66)
Medical leave/retired	37.4 (46)
Unemployed	7.3 (9)
Student	1.6 (2)
Marital status	
Married/living together	63.4 (78)
Divorced	13.8 (17)
Single	21.1 (26)
Widowed	1.6 (2)
Digital literacy (self-rated)	
Low	1.6 (2)
Acceptable	13.0 (16)
Good	38.2 (47)
Very good	47.2 (58)
Primary diagnosis	
Breast	76.4 (94)
Hematological	8.9 (11)
Gynecological	5.7 (7)
Other	8.9 (11)
Clinical status	
Post-cancer treatment	39.0 (48)
Undergoing adjuvant treatment	61.0 (75)
Sleeping pills	
Never	45.5 (56)
No, < 2 nights/week	17.9 (22)
Yes, $\ge$ 2 nights/week	10.6 (13)
Yes, everyday	26.0 (32)
Sleeping pill category (*n* = 45)	
Benzodiazepine	55.6 (25)
Antidepressant	37.8 (17)
Melatonin	11.1 (5)
Z drug	11.1 (5)
Over the counter	4.4 (2)

### Acceptability

**Fig. 1:**
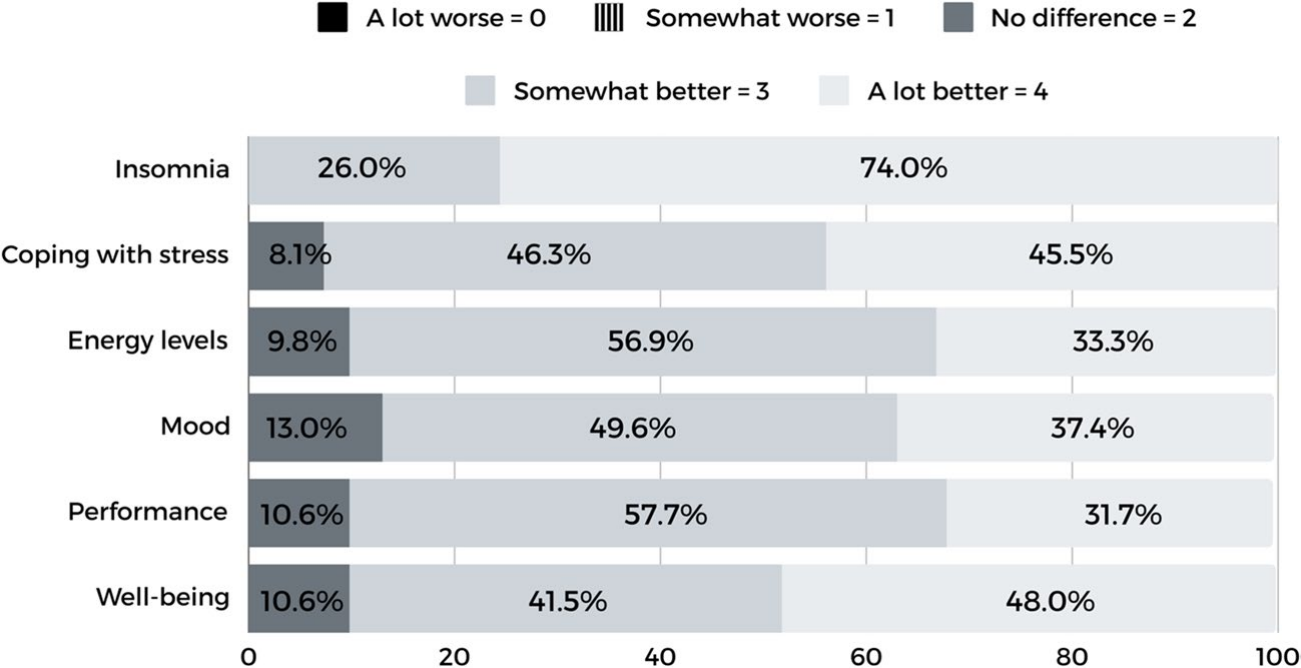
Perceived efficacy of digital CBT-I on insomnia and daytime outcomes

All participants indicated that the digital intervention helped their insomnia to become “a lot better” (74.0%) or “somewhat better” (26.0%; Fig. 1). Participants generally perceived the digital CBT-I program as very effective, with a mean self-reported satisfaction regarding perceived efficacy score of 4.4 out of 5.0 (95% CI: 3.2–5.0).

**Fig. 2:**
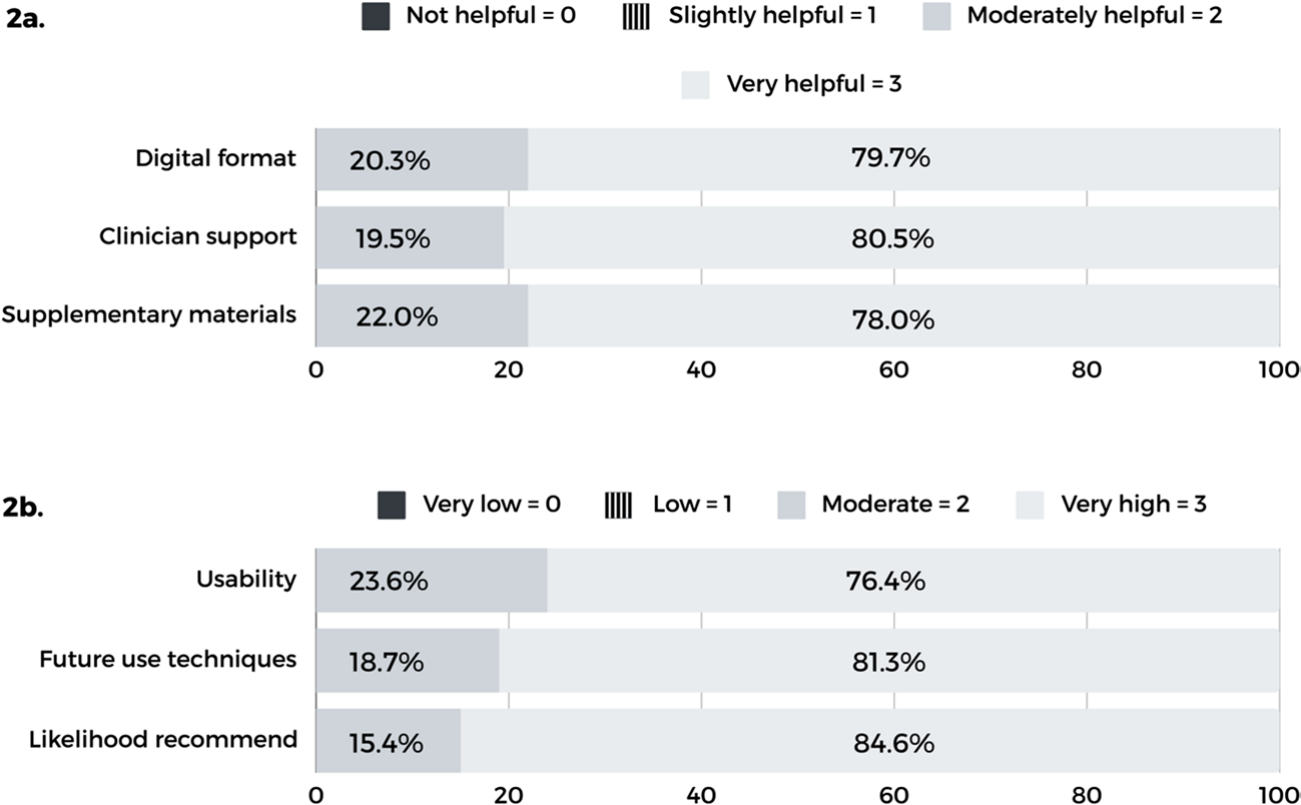
Perceived helpfulness of digital psychological intervention’s characteristics and ratings of usability, likelihood of future use of therapeutic techniques, and of recommending the digital intervention to a friend facing a similar problem. **2a** Clinician support included weekly feedback from the therapist via email. Supplementary materials, such as educational handouts, audio recordings, and planners, were provided weekly after each session. Higher scores denote greater perceived helpfulness of each category. **2b** Higher scores indicate better perceived usability, greater probability of continued use, and a stronger likelihood of recommending the intervention

All participants ranked clinician support, format, and material to be “very helpful” or “moderately helpful” (Fig. 2). In terms of overall satisfaction, participants were “very satisfied” (84.6%) or moderately satisfied (15.4%) with OncoSleep. No significant differences were found in overall satisfaction and perceived efficacy based on self-rated digital literacy; clinical, occupational or marital status, age group, or education level.

The overall score on the UEQ-S was 2.8 (SD = 0.3, CI = 2.7–2.9). The pragmatic quality scale score was 2.9 (SD = 0.2, CI = 2.8–3.0), while the hedonic quality scale was 2.7 (SD = 0.4, CI = 2.6–2.8). SDs in the UEQ-S show high agreement between participants concerning user-experience. A better user experience was associated with greater improvements in insomnia severity following digital CBT-I [*r* = − 0.26, *n* = 66, *p* = 0.033]. Participants who showed clinically significant improvements in insomnia severity reported better user experience [*X*^*2*^ = 19.6, *p* = 0.01] compared to those who did not.

### Experiences with implementing digital CBT-I: main themes

Free-text response feedback was provided by 104 cancer survivors. Participants’ insights reflected satisfaction with the intervention, as evidenced by responses such as, “The program was fantastic,” “I am very happy with the results,” and “I really enjoyed the program.” Eight themes were identified regarding factors influencing engagement with treatment (Table 4).

**Table 4:** Overview of themes, summary, and example responses

Theme	Description	Examples
Appreciated the clinician support and monitoring	Free responses categorized under this theme focused on the guidance and feedback provided by the therapist throughout the intervention, including codes related to a sense of connection, personalized support, and ongoing clinician monitoring	“I was always looking forward to the weekly email from the therapist with my feedback” “I’m really proud of how far I’ve come, and I know I wouldn’t have made these strides without the clinician’s help guiding me through the treatment” “I felt safe because a specialist was monitoring my progress”
Perceived treatment techniques as effective (but challenging)	This theme consisted of free responses related to specific therapeutics, covering both those that patients liked and found effective, as well as those they struggled to implement. Subthemes included improvements in sleep, generalized improvements, shifts in perspective, the need for commitment, challenges in implementing techniques, and skill development	“I now use a strategy that amuses me a lot with is trying to stay awake instead of trying hard to sleep. It’s great fun because it actually works and shows you how your mindset can really impact your body’s natural processes” “Even though some of the habits were already part of my routine, at times, I felt like there were too many strategies to integrate, and I struggled to keep up” “From my experience, those relaxation exercises helped my night sweats, which were totally throwing off my sleep and getting me really frustrated”
Interactivity and ease of use of the intervention felt engaging and motivating	This theme focused on navigation, content format, and the features of the digital CBT-I platform, including the intervention tools, user interface, and user experience subthemes	“I really liked building my sleep window (…) It felt great to see my sleep get better each week (…) and the progress showing up in the charts” “The information is concise and clear, and the illustrations and visuals always put a smile on my face” “It would be helpful if the materials where you can write were editable directly within the program. (…) It would be easier to use if the documents were directly editable”
Enjoyed the convenience of the digital intervention and valued the non-hospital setting	This theme focused on the characteristics of the treatment, particularly its online format and accessibility that facilitated participant engagement, with an emphasis on flexibility and accessibility. Subthemes included revisiting content, flexible scheduling, and home-based intervention	“(…) being able to check back whenever I needed to. It’s super helpful because I tend to forget what doctors say during appointments, especially since treatment left me with memory issues. Having all that info online really helps” “I don’t like going back to the hospital where I was treated, so receiving treatment online was perfect for me”
Considered the digital intervention as fulfilling unmet needs	This theme captured the feeling that the digital intervention addressed a gap experienced by cancer survivors. Subthemes included a sense of being understood and supported, as well as the need for comprehensive support for post-treatment needs	“What I liked most was that this program was easy for me to access and provided direct access to a sleep expert who could address questions and concerns that no one in the hospital was able to help with” “Few people understand the struggle of not being able to sleep properly, and in OncoSleep I found the support and guidance I needed”
Connected to tailored survivorship-content	This theme captured participants’ feedback on the personalization of treatment content to their cancer-related needs, including sensitivity to clinical complexities and shared stories	“So many times I could see myself in the examples of other cancer survivors” “I related a lot to the stories from other survivors, and it helped me realize I had some fears about the cancer coming back that I didn’t even know I had”
Preference for non-pharmacological treatment and discontinuation of medication	This theme featured free-text responses focused on insomnia medication, including lack of medication guidance and medication tapering	“I did not know this psychological treatment existed before but I’m so happy because I’m sleeping well, waking up refreshed, and most importantly, I accomplished this without medication – just by using the strategies I’ve learned” “My goal was to get off medication, but the program did not include advice on that” “It requires commitment, but I’ve been able to reduce my medication by half”
Felt empowered	This theme illustrates how patients developed confidence and agency, including a sense of autonomy, self-awareness, and self-management skills	“I’ve realized that I have the power to not let bad nights of sleep or night sweats ruin my day and because of this I feel like I’m truly enjoying my days now, something I haven’t felt in a long time” “I not only learned a lot about my sleep but also about myself, which has improved my overall quality of life”

#### Appreciated the clinician support and monitoring

Participants consistently valued clinician support as a central element of their treatment, helping them feel connected, safe, and motivated to engage with the program. They expressed appreciation for having a professional track their sleep patterns, respond to inquiries, and offer tailored guidance. Although the clinician feedback was delivered remotely via email, participants felt a strong sense of attentiveness: “(…) I never felt any lack of connection compared to in-person appointments.”

#### Perceived treatment techniques as effective (but challenging)

In discussing the aspects of treatment they liked least, patients shared their difficulties with implementing certain treatment techniques, which varied from person to person. For most, sleep restriction therapy was particularly challenging, while others struggled with relaxation techniques or stimulus control instructions. Two patients noted that the cognitive strategies initially heightened their anxiety. Nevertheless, participants recognized the value of these techniques; for example, some reported finding sleep restriction therapy (referred to as sleep consolidation in a sleep window prescription) hard to implement at first but acknowledged its effectiveness (“There were challenging moments, like adhering to my first sleep windows, but the improvements came quickly,” “Initially, I was apprehensive about reducing my time in bed, but I now fully appreciate the importance of this strategy”).

A common sentiment among participants was that the most beneficial aspect of the treatment was the change in their perspective on insomnia, as illustrated by: “I’ve taken insomnia off the center stage in my life, and this shift in perspective has been my greatest achievement and source of strength,” “(…) not obsessing over sleep, that strategy really works! It’s such a relief not to spend the whole day worrying about whether I’ll sleep or not.” Participants noted that while the therapeutic techniques are effective, they require motivation and commitment (“This treatment requires commitment,” “I’d say it takes effort and can be tough at times, but it’s so worth it. I feel way more energized and I enjoy my days more now”), and some reported to struggle to implement the therapeutics (e.g., “I wasn’t able to do all the strategies”). Patients enjoyed having a range of strategies at their disposal and noted that they could continue using them over time (e.g., “What I value most is that these strategies are not just temporary fixes but are tools I can use long-term”). Some participants observed that the intervention produced meaningful effects despite its brief duration (e.g., “My quality of life has improved after just six sessions”). The impact of sleep education was also evident across participants’ responses (e.g., “I appreciated learning about how insomnia develops after a cancer diagnosis and the various factors involved. It would have been helpful to have this information beforehand […]”).

#### Interactivity and ease of use of the digital intervention felt engaging and motivating

Participants often described sessions as “clear and accessible” and the platform “easy to navigate,” emphasizing its user-friendly nature. Participants particularly enjoyed tracking their progress throughout the intervention weeks through weekly stats and graphs, as well as monitoring their sleep using the interactive sleep diary (e.g., “I was always checking my weekly stats to see how I was doing and the truth is that you really feel improvements as you go”). Negative feedback on engagement included suggestions for incorporating more built-in features into the digital intervention, such as features to increase engagement with between-session activities (e.g., directly log their though-monitoring records and behavioral experiments, keep digital activity diaries/planning, and tools to improve communication with the clinician). Three participants also expressed a preference for a mobile app in addition to the web-based version. A participant expressed having seen some information before, while another expressed feeling overwhelmed due to the number of strategies introduced during the intervention.

#### Enjoyed the convenience of the digital intervention and valued the non-hospital setting

The online format was perceived as practical and suitable, allowing participants to surpass barriers such as living in remote areas, travel difficulties, and family or work responsibilities. Participants valued the ability to attend the modules at times that fit their schedules and being able to revisit treatment content as needed.

Notably, several participants expressed a preference for remote follow-up, stating that they preferred not to return to the hospital, as they aimed to maintain a sense of normalcy in their lives (e.g., “I loved that the program was online, yet I still had support whenever I needed it. The days I have to return to the [hospital] for appointments can be so nerve-wracking”; “For me, the fact that it wasn’t delivered in a hospital setting was ideal as I don’t like to see myself as a cancer survivor”). A few patients remarked that digital interventions could be a helpful means for people with insomnia to receive this treatment, as the stigma attached to visiting mental health services in person may deter them from seeking help.

#### Digital intervention fulfilled and validated unmet needs

This digital solution offered them a sense of validation for their sleep disturbances, as illustrated by responses as “I used to feel like no one really understood how hard it is to not be able to sleep, and sometimes people just didn’t take it seriously, but sleep is so important for healing”; “I used to tell doctors about my sleep difficulties, but they would just say it was normal and not offer any help. Now I get all the different things that contribute to insomnia and how I can tackle it. I just wish this kind of support was available to everyone.” Many participants expressed a desire for follow-up sessions and more digital interventions to address other ongoing needs as part of their recovery (e.g., perceived cognitive impairment, sexual difficulties).

#### Connected to tailored survivorship-content

Participants valued how the material addressed the link between sleep disturbances and cancer, as well as strategies for managing treatment-related side effects that could impact sleep. They also resonated with “stories from other cancer survivors” (i.e., case vignettes).

#### Preference for non-pharmacological treatment and discontinuation of medication

Although the treatment did not address medication tapering, many participants mentioned they would have liked to or were able to reduce or discontinue their insomnia medication. Negative feedback included criticism about the absence of medication tapering guidance. Participants also highlighted the efficacy of digital CBT-I compared to pharmacotherapy, describing it as an effective and natural option free from side effects: “I realized the pills were not making me sleep, but reducing my anxiety of not sleeping, but with this treatment I feel better and healthier.”

#### Felt empowered

Participants noted that the information provided and a change in perspective allowed them to better understand themselves (e.g., “The most important thing for me was learning that how I cope with these challenges and how I approach sleep is within my control”). They felt more equipped with the skills necessary to overcome their sleep difficulties: “Unfortunately, I haven’t been able to see much improvement and still rely on sleeping pills. However, I now have a better understanding of my sleep cycles and how to manage the negative thoughts that come at night and I will continue using the techniques I learned because I believe I will improve.”

## Discussion

Insights gained through quantitative and qualitative data suggest guided digital CBT-I is well-accepted for cancer survivors. Triangulation of patient ratings and comments suggests overall satisfaction with the program, with a high percentage of patients recommending treatment to others and intending to continue to use the techniques. This aligns with the emergence of an empowerment theme, as participants valued the development of self-management skills they could sustain. Our findings echo previous research in which cancer survivors rated survivorship-tailored digital CBT-I highly for overall satisfaction, enjoyment, and continued use of the skills learned [37, 38]. Quantitative findings indicate that the digital psychological intervention was perceived as effective in improving insomnia and, for most patients, daytime functioning. This is consistent with patients’ feedback, where a recurring theme was the perceived effectiveness of the techniques. However, for a relevant proportion of survivors, digital CBT-I made no difference in coping with stress, energy levels, mood, performance, and well-being, suggesting that some cancer survivors may benefit more from a more comprehensive approach or additional interventions, as CBT-I primarily focuses on insomnia.

Greater treatment improvements in insomnia were associated with a better user experience with the digital intervention, highlighting the importance of user-friendly design and functionality for the success of digital CBT-I interventions. Qualitative insights further suggest that the user interface and interactive features are facilitators of treatment engagement. Seeing their progress weekly toward their goal through graphs and charts showing sleep indicators (e.g., total sleep time, sleep efficiency), along with an increased sleep window, appears to reinforce patients’ progress and provide them with a sense of control over their sleep, contributing to continued engagement with the digital intervention and acceptance and commitment to treatment techniques. Findings also suggest digital CBT-I programs may be effective in engaging patients in self-monitoring (e.g., completing sleep diaries online might be more convenient and motivating than traditional written worksheets). Consistently, a barrier to engagement with treatment included patients’ desire for more integrated features within the digital platform (e.g., fillable digital worksheets and in-app communication tools). Another barrier was type and extent of content provided. These barriers highlight several opportunities for improving digital psychological interventions to increase engagement. Potential improvements include integrating dynamic and clinician communication tools into treatment platforms, such as interactive worksheets, planners, and chat functions. As technology continues to evolve, digital interventions must adapt. Considering the suggestions for more interactive features, incorporating gamification elements (e.g., points and rewards systems, goal-based challenges) could further improve user engagement. Additionally, tailoring the type and depth of content to users’ needs could further aid engagement, including providing optional modules that adjust based on digital and health literacy levels (e.g., offering less redundant content to those with higher literacy and more support to those who need it).

The lack of information on medication tapering also emerged as a barrier. This suggests the potential for including an optional module on sleep medication titration in digital CBT-I programs, ideally with communication to the prescribing clinician, ensuring that patients who wish to reduce their medication have access to evidence-based strategies. Given the risks associated with psychotropic medication use, leveraging digital CBT-I to assist motivated patients in tapering their medication could help improve their health and quality of life. This does not imply that all patients with insomnia should discontinue their sleep medication; any decision regarding medication should be based on personal preference and clinical judgment and should reflect a collaborative process between the patient and the prescriber. Patients should always consult their doctor if they wish to taper off medication. Nevertheless, many patients referred for CBT-I are often on medication due to the limited availability of CBT-I. Qualitative findings indicate cancer survivors prefer digital CBT-I over sleep medication, viewing it as more effective, particularly in improving the daytime effects of insomnia. In cancer survivors, the use of sleep medications is associated with diminished quality of life, increased symptom burden, and potential drug interactions [39, 40]. Cancer survivors can also feel burdened by multiple medications, which may further motivate them to discontinue sleep medications. When compared to pharmacotherapy, CBT-I offers greater efficacy and durability in treatment outcomes, along with a much safer and more favorable side-effect profile.

Clinical support and monitoring emerged as a central facilitator of patients’ experiences with digital CBT-I, which aligns with ratings indicating that most participants found clinician support to be very helpful. This supports previous research highlighting that cancer survivors especially valued the clinician support in digital CBT-I [38]. Therapist feedback may be particularly valuable for cancer survivors, as ongoing support from their cancer care team often decreases in the post-treatment stage of the cancer journey, which can leave survivors feeling unsupported as they cope with significant cancer-related psychosocial symptom burden and attempt to achieve a sense of normalcy [41]. Incorporating clinician support can foster reassurance and trust, address individual needs, provide positive reinforcement and corrective feedback, thereby improving self-efficacy. Because CBT-I is a structured, technique-driven intervention, empathy and fostering a supportive environment through clinician feedback is likely to maximize engagement, patient acceptability, and outcomes. This is consistent with a meta-analysis reporting that guided digital interventions are associated with higher engagement and tend to be more effective than non-guided interventions for cancer survivors [42]. For insomnia, the addition of minimal clinician support to automated digital CBT-I is likely to be cost-effective, as the time commitment from the therapist for the entire treatment course is comparable to that of one in-person appointment [43].

Convenience also emerged as a facilitator of treatment engagement, aligning with quantitative results showing that all participants found the digital format to be moderately or very helpful. Participants stressed the value of having 24/7 access to treatment. This suggests that digital CBT-I programs can act as beneficial adjuncts to in-person CBT-I, facilitating patients’ ability to implement techniques effectively between sessions. Patients expressed a desire for follow-up sessions, highlighting the need for research into the impact of adding these sessions to digital interventions. Feedback from patients also indicates that the digital intervention addressed an important need that had not been met through traditional healthcare, sparking interest in further digital solutions for other cancer-related conditions. This underscores the potential of digital medicine in supportive cancer care, where digital interventions could be integrated into a survivorship care plans within an interconnected digitally enabled healthcare system [9]. Patients also highlighted the importance of sleep education, with several suggesting it would be helpful to develop this knowledge earlier. These findings point to the potential of early psychoeducational interventions to prevent cancer-related insomnia. Tailoring treatment content to the unique experiences of cancer survivors also appears to facilitate treatment engagement, consistent with previous research [23]. By addressing cancer-specific beliefs and side effects that can hinder adherence to CBT-I techniques, and by accommodating these techniques to their clinical complexities, patients may foster greater confidence in their treatment, perceive it as more adequate, and subsequently improve engagement, leading to better treatment outcomes.

A key limitation of this study is the overrepresentation of women in our sample, who were predominantly young, highly educated, and digitally literate. This reflects a pattern seen in other research on CBT-I for cancer survivors [44], potentially reflecting the disproportionate impact of insomnia on women, with breast cancer serving as a known risk factor. Moreover, digital interventions are likely more appealing to younger cancer survivors, while older individuals may encounter difficulties with engagement. However, this limits the generalizability of our findings to men and individuals with different educational backgrounds, who may be less likely to adopt these programs. To overcome this, future research should aim to recruit more diverse samples. Additionally, the sample may be biased toward more positive results, as it only includes treatment completers. It is possible that some participants who dropped out did so due to dissatisfaction, with the possibility that they may have found the techniques ineffective or too challenging, as these factors were identified as influencing treatment engagement. To address this important limitation, future studies can conduct implementation interviews with relevant stakeholders, including a diverse range of user groups—ranging from those who do not engage to those who complete all aspects of the intervention—to fully understand the barriers and facilitators affecting digital CBT-I engagement and acceptability. Other limitations include the reliance on a skilled clinician and the potential bias introduced by self-referral, which may reflect a higher motivation to engage with the treatment. Relying on experts to deliver the intervention requires training and resources, which limits its scalability. On the other hand, this model allows experts to reach a larger number of patients, thereby supporting greater scalability. Notwithstanding these limitations, to our knowledge, this study is among the first to explore the acceptability and experiences of cancer survivors using automated digital CBT-I with clinician support. Our findings provide evidence that digital CBT-I with clinician support is acceptable for cancer survivors, while also identifying barriers and facilitators to engagement with the intervention.

Self-administered digital interventions with clinician support widen access to guideline informed care and can work synergistically with specialized remote monitoring while empowering survivors with self-management skills. Digital CBT-I can be particularly relevant to meet the unique needs of cancer survivors, as it is trauma-informed (reducing the stress of returning to cancer centers) and considerate of illness burden constraints (e.g., comorbidities and complications, other treatment-related toxicities, and late effects). By increasing convenience, it can also address financial toxicity (e.g., benefiting patients who are unable to leave work).

## Data Availability

The data that support the findings of this study are available from the corresponding author upon reasonable request.

## References

[1] Garland SN (2022) Chapter 12 - CBT-I during and after a cancer diagnosis. In: Nowakowski S, Garland SN, Grandner MA, Cuddihy LJ (eds) Adapting cognitive behavioral therapy for insomnia. Academic Press, 235–264

[2] Cleeland CS, Zhao F, Chang VT et al (2013) The symptom burden of cancer: evidence for a core set of cancer-related and treatment-related symptoms from the Eastern Cooperative Oncology Group Symptom Outcomes and Practice Patterns study. Cancer 119(24):4333–4340. 10.1002/cncr.28376 24114037 10.1002/cncr.28376 PMC3860266

[3] Hong F, Blonquist TM, Halpenny B, Berry DL (2016) Patient-reported symptom distress, and most bothersome issues, before and during cancer treatment. Patient Relat Outcome Meas 7:127–135. 10.2147/PROM.S95593 27672346 10.2147/PROM.S95593 PMC5026183

[4] Savard J, Ivers H, Villa J, Caplette-Gingras A, Morin CM (2011) Natural course of insomnia comorbid with cancer: an 18-month longitudinal study. J Clin Oncol 29(26):3580–3586. 10.1200/JCO.2010.33.2247 21825267 10.1200/JCO.2010.33.2247

[5] Lowery-Allison AE, Passik SD, Cribbet MR et al (2018) Sleep problems in breast cancer survivors 1–10 years posttreatment. Palliat Support Care 16(3):325–334. 10.1017/S1478951517000311 28508735 10.1017/S1478951517000311

[6] American Psychiatric Association (2013) Diagnostic and statistical manual of mental disorders, 5th edn. American Psychiatric Publishing, Arlington

[7] Reynolds-Cowie P, Fleming L (2021) Living with persistent insomnia after cancer: a qualitative analysis of impact and management. Br J Health Psychol 26(1):33–49. 10.1111/bjhp.12446 32558129 10.1111/bjhp.12446

[8] Howell D, Oliver TK, Keller-Olaman S et al (2013) A Pan-Canadian practice guideline: prevention, screening, assessment, and treatment of sleep disturbances in adults with cancer. Support Care Cancer 21(10):2695–2706. 10.1007/s00520-013-1823-6 23708820 10.1007/s00520-013-1823-6

[9] Clara MI, Canavarro MC, Miller-Mendes M, Allen GA (2023) Insomnia in cancer survivors: a precision behavioral sleep medicine approach. European Psychol 28(2):110–112. 10.1027/1016-9040/a000506

[10] Léger D, Scheuermaier K, Philip P, Paillard M, Guilleminault C (2001) SF-36: evaluation of quality of life in severe and mild insomniacs compared with good sleepers. Psychosom Med 63(1):49–55. 10.1097/00006842-200101000-00006 11211064 10.1097/00006842-200101000-00006

[11] Irwin MR (2013) Depression and insomnia in cancer: prevalence, risk factors, and effects on cancer outcomes. Curr Psychiatry Rep 15(5):404. 10.1007/s11920-013-0404-1 24078066 10.1007/s11920-013-0404-1 PMC3836364

[12] Ruel S, et al (2017) Insomnia, immune functioning and infections in cancer patients treated with chemotherapy: results from a longitudinal study. Presented at: 38th Annual Meeting of the Society of Behavioral Medicine; March 2017; San Diego, California

[13] Savard J, Ivers H, Savard MH et al (2021) Efficacy of a stepped care approach to deliver cognitive-behavioral therapy for insomnia in cancer patients: a noninferiority randomized controlled trial. Sleep 44(11):zsab166. 10.1093/sleep/zsab166 34228123 10.1093/sleep/zsab166 PMC8598200

[14] Grassi L, Zachariae R, Caruso R et al (2023) Insomnia in adult patients with cancer: ESMO Clinical Practice Guideline. ESMO Open 8(6):102047 38158225 10.1016/j.esmoop.2023.102047 PMC10774975

[15] Sanft T, Day A, Ansbaugh S et al (2023) NCCN Guidelines® Insights: Survivorship, Version 1.2023. J Natl Compr Canc Netw 21(8):792–803. 10.6004/jnccn.2023.0041 37549906 10.6004/jnccn.2023.0041

[16] Garneau J, Savard J, Dang-Vu TT, Gouin JP (2024) Predicting response to stepped-care cognitive behavioral therapy for insomnia using pre-treatment heart rate variability in cancer patients. Sleep Med 121:160–170. 10.1016/j.sleep.2024.06.021 38991424 10.1016/j.sleep.2024.06.021

[17] Zachariae R, Amidi A, Damholdt MF et al (2018) Internet-delivered cognitive-behavioral therapy for insomnia in breast cancer survivors: a randomized controlled trial. J Natl Cancer Inst 110(8):880–887. 10.1093/jnci/djx293 29471478 10.1093/jnci/djx293 PMC6093474

[18] Clara MI, van Straten A, Savard J, Canavarro MC, Allen Gomes A (2025) Web-based cognitive-behavioral therapy for insomnia in cancer survivors: the OncoSleep randomized trial. Sleep Med 129:67–74. 10.1016/j.sleep.2025.02.021 10.1016/j.sleep.2025.02.021 39987779

[19] Sekhon M, Cartwright M, Francis JJ (2017) Acceptability of healthcare interventions: an overview of reviews and development of a theoretical framework. BMC Health Serv Res 17(1):88. 10.1186/s12913-017-2031-8 28126032 10.1186/s12913-017-2031-8 PMC5267473

[20] Hommel KA, Hente E, Herzer M, Ingerski LM, Denson LA (2013) Telehealth behavioral treatment for medication nonadherence: a pilot and feasibility study. Eur J Gastroenterol Hepatol 25(4):469 23325274 10.1097/MEG.0b013e32835c2a1b PMC3703848

[21] Schrepp M, Hinderks A, Thomaschewski J (2014) Applying the User Experience Questionnaire (UEQ) in different evaluation scenarios. In: Marcus A, ed. Design, user experience, and usability: theories, methods, and tools for designing the user experience. Vol 8517. Lecture Notes in Computer Science. Springer International Publishing; 383–392.

[22] Luik AI, Kyle SD, Espie CA (2017) Digital cognitive behavioral therapy (dCBT) for insomnia: a state-of-the-science review. Curr Sleep Med Rep 3(2):48–56. 10.1007/s40675-017-0065-4 28553574 10.1007/s40675-017-0065-4 PMC5427093

[23] Cheng P, Santarossa S, Kalmbach D, Sagong C, Hu K, Drake C (2023) Patient perspectives on facilitators and barriers to equitable engagement with digital CBT-I. Sleep Health 9(5):571–578. 10.1016/j.sleh.2023.07.003 37625947 10.1016/j.sleh.2023.07.003 PMC10592026

[24] Middlemass J, Davy Z, Cavanagh K et al (2012) Integrating online communities and social networks with computerised treatment for insomnia: a qualitative study. Br J Gen Pract J R Coll Gen Pract 62(605):e840-850. 10.3399/bjgp12X659321 10.3399/bjgp12X659321 PMC3505418 23211265

[25] Pramana G, Parmanto B, Lomas J, Lindhiem O, Kendall PC, Silk J (2018) Using mobile health gamification to facilitate cognitive behavioral therapy skills practice in child anxiety treatment: open clinical trial. JMIR Serious Games 6(2):e8902. 10.2196/games.8902 10.2196/games.8902 PMC5968217 29748165

[26] Zhou ES, Ritterband LM, Bethea TN, Robles YP, Heeren TC, Rosenberg L (2022) Effect of culturally tailored, internet-delivered cognitive behavioral therapy for insomnia in black women: a randomized clinical trial. JAMA Psychiat 79(6):538–549. 10.1001/jamapsychiatry.2022.0653 10.1001/jamapsychiatry.2022.0653 PMC9021979 35442432

[27] Mills N, Donovan JL, Wade J, Hamdy FC, Neal DE, Lane JA (2011) Exploring treatment preferences facilitated recruitment to randomized controlled trials. J Clin Epidemiol 64(10):1127–1136. 10.1016/j.jclinepi.2010.12.017 21477994 10.1016/j.jclinepi.2010.12.017 PMC3167372

[28] Clara MI, van Straten A, Canavarro MC, Allen GA (2024) Digital cognitive-behavioral therapy for insomnia in cancer survivors: protocol for a pragmatic clinical trial. Acta Med Port 37(10):713–719. 10.20344/amp.16344 39140169 10.20344/amp.21094

[29] Morin CM, Espie CA (2013) Insomnia: a clinical guide to assessment and treatment. Springer, New York

[30] Seligman MEP (1995) The effectiveness of psychotherapy: the consumer reports study. Am Psychol 50:965–974 8561380 10.1037//0003-066x.50.12.965

[31] Manber R, Bernert RA, Suh S, Nowakowski S, Siebern AT, Ong JC (2011) CBT for insomnia in patients with high and low depressive symptom severity: adherence and clinical outcomes. J Clin Sleep Med 7(6):645–652. 10.5664/jcsm.1472 22171204 10.5664/jcsm.1472 PMC3227711

[32] Moloney ME, Dunfee M, Rutledge M, Schoenberg N (2020) Evaluating the feasibility and acceptability of internet-based cognitive behavioral therapy for insomnia in rural women. Women’s Health Report 114–122. 10.1089/whr.2020.0053. 10.1089/whr.2020.0053 PMC7325489 32617531

[33] Schrepp M, Hinderks A, Thomaschewski J (2017) Design and evaluation of a short version of the User Experience Questionnaire (UEQ-S). IJIMAI 4(6):103–108

[34] Clemente V, Ruivo Marques D, Miller-Mendes M, Morin CM, Serra J, Allen GA (2021) The European Portuguese version of the insomnia severity index. J Sleep Res 30(1):e13198. 10.1111/jsr.13198 32997368 10.1111/jsr.13198

[35] Braun V, Clarke V (2006) Using thematic analysis in psychology. Qual Res Psychol 3(2):77–101. 10.1191/1478088706qp063oa

[36] QSR International (2018) NVivo 12 (Version 12) [Computer software]. QSR International

[37] Hall DL, Arditte Hall KA, Gorman MJ et al (2022) The Survivorship Sleep Program (SSP): a synchronous, virtual cognitive behavioral therapy for insomnia pilot program among cancer survivors. Cancer 128(7):1532–1544. 10.1002/cncr.34066 34914845 10.1002/cncr.34066 PMC8917089

[38] Dozeman E, Verdonck-de Leeuw IM, Savard J, van Straten A (2017) Guided web-based intervention for insomnia targeting breast cancer patients: feasibility and effect. Internet Interv 9:1–6. 10.1016/j.invent.2017.03.005 30135831 10.1016/j.invent.2017.03.005 PMC6096207

[39] Paltiel O, Marzec-Boguslawska A, Soskolne V et al (2004) Use of tranquilizers and sleeping pills among cancer patients is associated with a poorer quality of life. Qual Life Res 13(10):1699–1706. 10.1007/s11136-004-8745-1 15651540 10.1007/s11136-004-8745-1

[40] Kelly CM, Juurlink DN, Gomes T, Duong-Hua M, Pritchard KI, Austin PC, Paszat LF (2010) Selective serotonin reuptake inhibitors and breast cancer mortality in women receiving tamoxifen: a population-based cohort study. BMJ 340:c693. 10.1136/bmj.c693 10.1136/bmj.c693 PMC2817754 20142325

[41] National Research Council, Institute of Medicine (2006) From cancer patient to cancer survivor: lost in transition: an American Society of Clinical Oncology and Institute of Medicine Symposium. Washington, DC: National Academies Press

[42] Akdemir A, Smith AB, Wu VS et al (2024) Guided versus non-guided digital psychological interventions for cancer patients: a systematic review and meta-analysis of engagement and efficacy. Psychooncology 33(1):e6290. 10.1002/pon.6290 38282223 10.1002/pon.6290

[43] Lancee J, van den Bout J, Sorbi MJ, van Straten A (2013) Motivational support provided via email improves the effectiveness of internet-delivered self-help treatment for insomnia: a randomized trial. Behav Res Ther 51(12):797–805. 10.1016/j.brat.2013.09.004 24121097 10.1016/j.brat.2013.09.004

[44] Ritterband LM, Bailey ET, Thorndike FP, Lord HR, Farrell-Carnahan L, Baum LD (2012) Initial evaluation of an Internet intervention to improve the sleep of cancer survivors with insomnia. Psychooncology 21(7):695–705. 10.1002/pon.1969 21538678 10.1002/pon.1969 PMC3424270

